# The combined effects of sediment accretion (burial) and nutrient enrichment on the growth and propagation of *Phalaris arundinacea*

**DOI:** 10.1038/srep39963

**Published:** 2017-01-05

**Authors:** Xinsheng Chen, Yulin Liao, Yonghong Xie, Chao Wu, Feng Li, Zhengmiao Deng, Xu Li

**Affiliations:** 1 Key Laboratory of Agro-ecological Processes in Subtropical Region, The Chinese Academy of Sciences, Hunan 410125, China; 2 Dongting Lake Station for Wetland Ecosystem Research, Institute of Subtropical Agriculture, The Chinese Academy of Sciences, Changsha 410125, China; 3 Soil and Fertilizer Institute of Hunan province, Hunan Academy of Agricultural Sciences, Changsha 410125, China

## Abstract

Sediment accretion (burial) and nutrient enrichment occur concurrently in lacustrine wetlands, but the role of these two aspects of sedimentation on macrophyte performance has rarely been examined. Here, we investigated the concurrent effects of sediment accretion and nutrient enrichment on the growth and propagation of *Phalaris arundinacea* L. using a factorial sediment burial by nutrient addition experimental design. Regardless of burial depth, nutrient addition increased biomass accumulation, shoot mass ratio, the number of rhizomes, and the length of ramets and rhizomes. While burial had little effect on plant growth and propagation, it had an interactive effect with nutrient addition on belowground growth and ramet production. These results indicate that *P. arundinacea* is tolerant to burial, which allows it to grow in habitats with high sedimentation rates. However, the enhanced growth and propagation of *P. arundinacea* following sedimentation were primarily related to nutrient enrichment. This suggests that nutrient enrichment of sediments, which occurs in many lacustrine wetlands, increases the risk of invasion by *P. arundinacea*.

Sedimentation is a common phenomenon in lacustrine wetlands that can be caused by changes in the water level, sediment input, and settlement of suspended particles^1^. The depth of sedimentation varies from a few millimeters to several centimeters per year, depending on watershed management practices^1^.

Sedimentation has a number of significant consequences for the biotic and abiotic environment^1^. In particular, burial by sediment is a major selective force on the growth and distribution of clonal macrophytes, which are a common feature of wetland habitats^1^,^2^. Wetland macrophytes may respond to burial by stem elongation, increased stem biomass, and release of dormant buds on their shoots and rhizomes to overcome soil mechanical resistance and grow above the sediment surface^3^,^4^,^5^,^6^. The phenomenon of shallow burial stimulating the growth of clonal macrophytes has been observed in tidal wetlands^7^, salt marshes^8^, and freshwater wetlands^3^. Nevertheless, wetland macrophytes generally cannot tolerate deep burial, as sand dune species can^1^,^3^,^4^.

Sedimentation increases the availability of nutrients for plant growth, as the recently accreted sediments act as sinks, sources, and transformers of the influent materials, which include nutrients and contaminants^9^. Nutrient availability is a key influence on biomass production, resource allocation, and reproductive strategy in clonal macrophytes^10^,^11^,^12^. Nutrient enrichment may increase the shoot length, total biomass, and shoot: root ratio of wetland macrophytes^10^,^12^. However, wetland macrophytes responses to sediment nutrient enrichment vary from none to positive^13^.

Moreover, the effects of sediment accretion and nutrient enrichment on plant performance might not be independent^14^,^15^. For example, increased nutrient supply may facilitate the acclimation of coastal dune species to sand accretion^14^,^16^. However, the concurrent effects of sediment accretion and nutrient enrichment on growth and propagation of clonal macrophytes have rarely been examined in lacustrine wetlands.

In the present study, we experimentally investigated the interaction between sediment accretion (burial) and nutrient enrichment on plant growth and propagation. To determine the role of these two aspects of sedimentation, we used a factorial design (sediment burial by nutrient addition) in *Phalaris arundinacea* L. (Poaceae; reed canarygrass), a widely distributed temperate northern hemisphere plant of marshes, rivers, and lake margins that are often disturbed by sedimentation^17^,^18^. *Phalaris arundinacea* has been extensively used as a forage and bioenergy crop, as well as in restoration of degraded soils and water^19^, and is notable as an invader of many natural wetlands in North America^17^,^19^. Previous work has indicated that it responds to moderate sediment burial by increasing its production of ramets^6^. Here, we test the following hypotheses: (1) both moderate sediment burial and nutrient addition will increase growth and propagation of *P. arundinacea*; (2) deep sediment burial will decrease its growth, while nutrient addition will ameliorate this negative effect.

## Results

### Biomass accumulation and allocation

**Figure 1:**
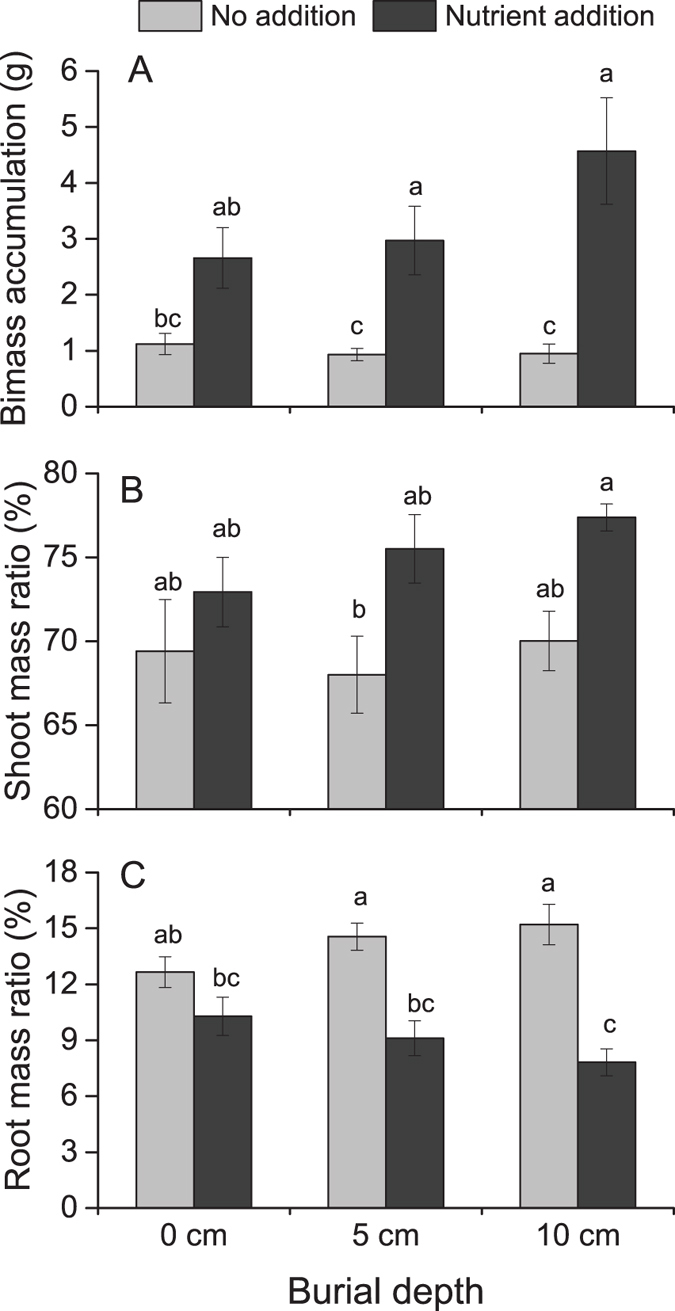
Biomass accumulation, shoot mass ratio, and root mass ratio in *Phalaris arundinacea* growing at three sedimentation depths with two nutrient levels. Standard error bars sharing the same lower case letters are not significantly different (*P* > 0.05).

Regardless of burial depth and nutrient level, 100% of the plants survived. Biomass accumulation and shoot mass ratio were significantly affected only by nutrient levels (Table 1; Fig. 1A,B), with no effect of burial depth. Nutrient addition increased biomass accumulation in plants buried at 5-cm and 10-cm depths (Fig. 1A). Shoot mass ratio was highest in plants at 10-cm burial depth with added nutrients and lowest at 5-cm burial depth without added nutrients. Root mass ratio, on the other hand, was not only affected by nutrient levels but also showed significant interacting effects of burial depth and nutrient level (Table 1; Fig. 1C), with nutrient addition decreasing root mass ratio at burial depths of 5 cm and 10 cm (Fig. 1C). Rhizome mass ratio was not affected by either burial depth or nutrient level (Table 1).

### Number of new ramets, rhizomes, and buds

**Figure 2:**
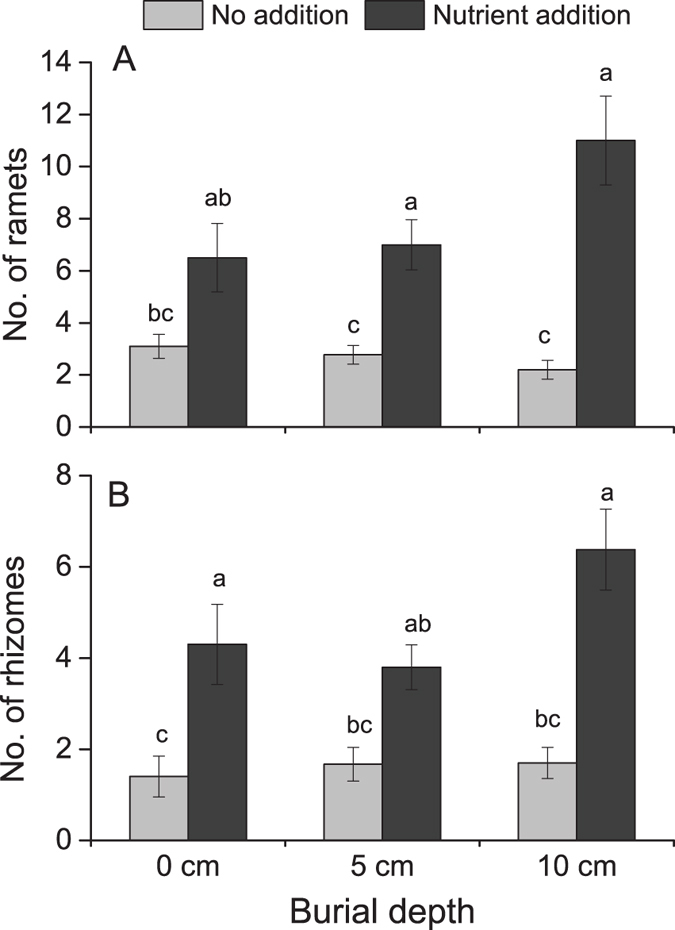
The number of ramets and rhizomes produced by *Phalaris arundinacea* growing at three sedimentation depths with two nutrient levels. Standard error bars sharing the same lower case letters are not significantly different (*P* > 0.05).

Nutrient level altered the number of new ramets produced by *P. arundinacea* and interacted with burial depth (Table 1; Fig. 2A). Nutrient addition increased the number of new ramets at 5-cm and 10-cm burial depths. The number of new rhizomes was only affected by nutrient level (Table 1; Fig. 2B), with nutrient addition increasing the number of new rhizomes at 0-cm and 10-cm burial depths. The number of buds was not affected by either burial depth or nutrient level (Table 1).

### Length of ramets, spacers, and rhizomes

**Figure 3:**
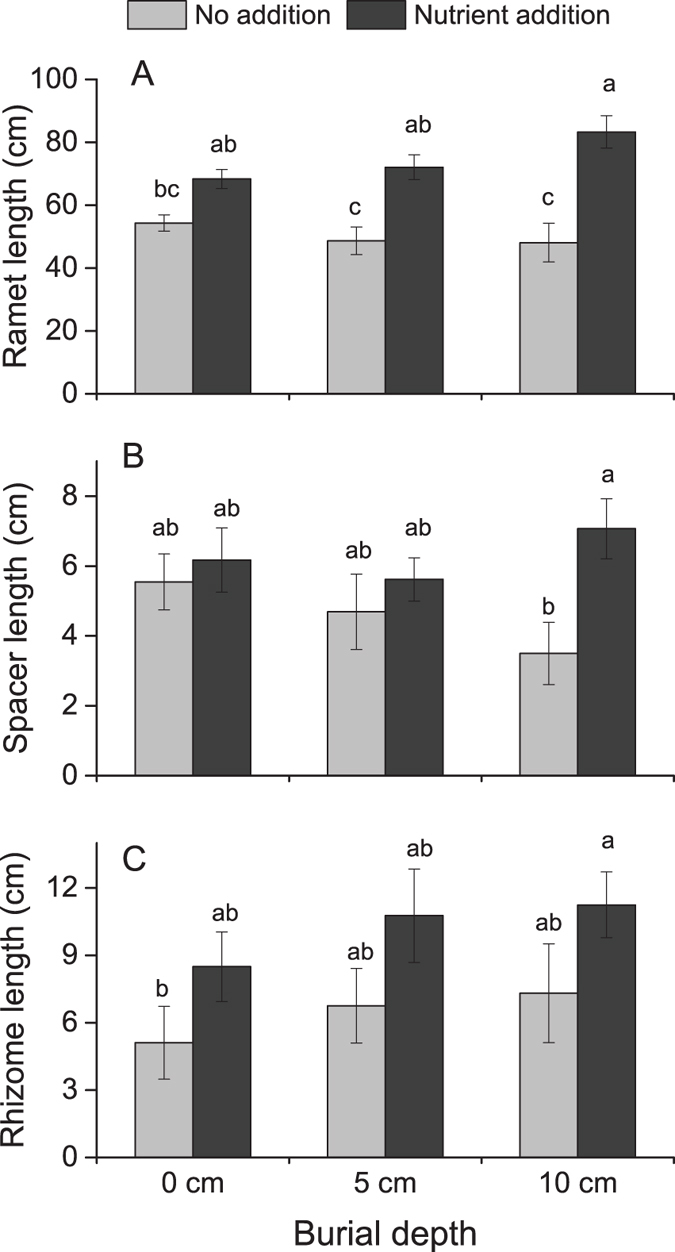
The length of ramets, spacers, and rhizomes of *Phalaris arundinacea* growing at three sedimentation depths with two nutrient levels. Standard error bars sharing the same lower case letters are not significantly different (*P* > 0.05).

The length of ramets, spacers, and rhizomes was only affected by nutrient level (Table 1, Fig. 3). Ramets were longer when nutrients were added at 5-cm and 10-cm burial depths (Fig. 3A). Spacers between ramets were longest when nutrients were added at 10-cm burial depth and shortest at the same burial depth without nutrient addition (Fig. 3B). New rhizomes were longest in plants at 10-cm burial depth with nutrient addition and shortest in plants at 0-cm burial depth without nutrient addition (Fig. 3C).

## Discussion

In this study, we conducted a factorial experiment to investigate the concurrent effects of sediment accretion and nutrient enrichment on the growth of a wetland plant, *Phalaris arundinacea*. Sediment accretion alone did not affect biomass accumulation, biomass allocation, or the number of propagules produced. This result is not consistent with our first hypothesis, which predicted that moderate sediment burial would stimulate plant growth. Sediment accretion reduces oxygen levels in the root zone; hypoxia is considered the major constraint on plant growth in lacustrine wetlands^20^,^21^,^22^. *Phalaris arundinacea* can acclimate to anoxia by growing thicker and shorter lateral roots and increasing soluble sugar contents^23^. Increased biomass accumulation and ramet production have been observed in *P. arundinacea* shortly following burial, probably over one or two months^6^,^23^. However, plants are less able to acclimate by three months, owing to a consistently decreasing carbohydrate content^23^. In our study, plants were buried for almost four months before being measured, so earlier positive responses to sediment accretion may have been missed.

Our finding that plant biomass and the production of ramets and rhizomes were not significantly reduced even under deep sediment burial (10 cm) further indicates that *P. arundinacea* is tolerant to burial. This contrasts with the general pattern that wetland macrophytes, unlike sand dune species, cannot tolerate high sedimentation rates^1^,^3^. In the Dongting Lake wetlands, for example, burial under 10 cm of sediment reduced plant biomass for three dominant emergent macrophytes (*Carex brevicuspis, Miscanthus sacchariflorus*, and *Polygonum hydropiper*)^3^,^4^. The unusual tolerance of *P. arundinacea* to sediment accretion may be related to its distribution^2^,^24^: in the Dongting Lake wetlands, it occurs primarily in the lower boundary of the vegetation zone, where the sedimentation rate is much higher than at sites higher up on the lake shore^2^. *Phalaris arundinacea* may therefore have evolved a variety of adaptations that allow survival and vegetative propagation in high-sedimentation habitats^6^,^17^,^23^.

Regardless of burial depth, nutrient addition increased biomass accumulation, shoot mass ratio, number of rhizomes, and the length of ramets and rhizomes. This suggests that the stimulation of growth of *P. arundinacea* in response to burial under sediment is caused primarily by nutrient enrichment. In a mesocosm experiment, Kercher and Zedler^17^ also indicated that nutrient-rich sediment (~ 5 cm layer of loamy topsoil) increased the biomass of *P. arundinacea*, whereas nutrient-poor sediment (fine mason sand) has no significant effect on the biomass of *P. arundinacea*. The positive growth response of *P. arundinacea* to increased nutrient input has been demonstrated in several studies^17^,^25^,^26^,^27^: it can take advantage of nutrient input through increased biomass production^17^, increased allocation to shoots^26^,^28^, and increased rates of clonal spread and tiller production^6^,^19^,^25^.

While burial had little impact on growth and propagation of *P. arundinacea*, it had an interactive effect with nutrient addition on belowground growth and ramet production. For sand dune species, nutrient enrichment generally increased plant performance up to moderate burial, but it had no or even a negative effect at a deep burial level^1^,^14^. However, for *P. arundinacea*, nutrient enrichment increased root mass ratio and ramet production under partially buried conditions, and no consistent increase in root mass ratio and ramet production was found with surface plantings. In addition to increasing soil resources such as nutrients and moisture, sedimentation also increases soil volume and the activity of mycorrhizal fungi. All of these factors may contribute to the enhanced growth of macrophytes^1^,^15^.

Altogether, these observations suggest that the growth and vegetative spread of *P. arundinacea*, a fast-growing and potentially invasive species, are primarily nutrient-limited in oligotrophic wetlands. However, sediment nutrient concentrations in many lacustrine wetlands have been increasing owing to regular input of agricultural runoff from surrounding farmed areas^29^. The results of our study imply that the removal of nutrients from inflow runoff and sediments might be an effective tool for managing wetlands that have been invaded by *P. arundinacea*.

## Conclusion

Our results demonstrate that the enhanced growth and propagation of *P. arundinacea* in response to burial by sediment is primarily caused by nutrient enrichment. Nutrient enrichment of sediments, which occurs in many lacustrine wetlands, may thus increase the risk of invasion by *P. arundinacea*^24^,^30^. Furthermore, we found *P. arundinacea* to be tolerant to sediment burial, which allows it to grow in habitats with a high sedimentation rate. In such habitats, *P. arundinacea* may outcompete burial-intolerant species and form a monoculture^17^,^19^.

## Methods

### Study species

*Phalaris arundinacea* (Poaceae) is a widely distributed wetland plant of the northern hemisphere. Its culms are reed-like, leafy, and 0.6–1.5 m in height^18^. It produces vigorous underground rhizomes, enabling it to spread aggressively^31^. At the study site in Hunan Province, China, *P. arundinacea* flowers and fruits from April to June^32^. However, seedlings are scarce in the field because the plant recruits mainly by producing vegetative ramets from rhizome buds^32^.

### Experimental design

The experiment was conducted on wild plants collected from Liumenzha Village in the East Dongting Lake wetlands on 17 May 2015. In an area of ~5 m^2^, plant fragments with rhizomes were dug up and transported to the Dongting Lake Station for Wetland Ecosystem Research, Chinese Academy of Sciences, Yueyang, Hunan Province, China. Rhizomes were planted at a depth of 10 cm in a nursery bed containing a soil/sand mixture. The soil was taken from the upper layer of the plant collection site and included 1.14 mg kg^−1^ total nitrogen and 0.37 mg kg^−1^ total phosphorus. After germination, on June 17, 100 similar-sized plants were selected, planted into individual plastic containers (27 cm height, 24 cm diameter) that were filled with 10 cm soil, and allowed to grow. On June 30, 60 containers with similar-sized plants (4–5 leaves and 19–25 cm in height) were selected for the experiment.

The experimental design was a randomized block with ten replicates, conducted in separate outdoor water tanks (200 cm × 200 cm × 100 cm) containing water at a depth of 10 cm. Three levels of sediment accretion (0, 5, and 10 cm) and two levels of nutrient (no addition and nutrient addition) were used, with each combination applied to one of the six plants in each tank. During the experiment, two types of sediments were prepared. Sand collected from Dongting Lake, which contained 0.02 mg kg^−1^ total nitrogen and 0.04 mg kg^−1^ total phosphorous, was used as the no-addition sediment. Nutrient addition sediment was made by mixing sand homogeneously with slow-release fertilizer (1.5 g slow-release fertilizer per kg sand). The slow-release fertilizer was 312 S Osmocote Exact (N-P-K, 15-9-11+2MgO, The Scotts Company, USA). Approximately 2.5 kg or 5.0 kg of sediment (corresponding to a layer of sediment ~5 cm or 10 cm deep), with or without nutrient addition, was added to each container for the 5-cm or 10-cm burial treatment. For treatment at 0-cm burial with nutrient addition, 1.5 g slow-release fertilizer was applied to the soil surface and covered with a thin layer of sand (less than 0.2 cm). A thin layer of sand was also added for the treatment at 0 cm burial without nutrient addition.

The water level in the tanks was maintained at 10 cm (0 cm for the plants), with tap water added as required and surplus water removed after rain. Plants were checked each week and new ramets marked with plastic tags. Meteorological data were recorded by an automatic weather station (Milos 520, Vaisala, Finland) located *ca.* 150 m away from the experiment. During the experiment (June 30–October 24, 2015), daily air temperature was 25.4 ± 3.3 °C (mean ± SD).

### Harvest and measurement

The plants were harvested 117 days after treatment. Plants with any live aboveground material were defined as alive. The plants were carefully excavated from the tanks to maintain the connections between ramets, cleaned with tap water, and transported to the laboratory for measurements. The number of ramets, rhizomes, and rhizome buds, and the length of new ramets, rhizomes, and spacers produced by each plant were recorded. Each plant was then separated into shoots, roots, and rhizomes. The biomass of each plant part was measured after drying at 80 °C for 48 h in an oven. Biomass accumulation was calculated as the total plant dry weight at the end of the experiment. Biomass allocation was calculated as a ratio of the mass of each plant part, *i.e.*, shoot, root, and rhizome, relative to the biomass of the total plant.

### Data analysis

The roles of sediment accretion and nutrient addition were assessed using a general linear model (GLM) in terms of their effects on biomass accumulation, biomass allocation, the number of new ramets, rhizomes, and buds, and the length of new ramets, spacers, and rhizomes. Multiple comparisons of means were performed using Tukey’s test at the 0.05 significance level; Bonferroni corrections for multiple comparisons were made as appropriate. Data were log^10^-transformed if necessary to reduce the heterogeneity of variances, and homogeneity was confirmed using Levene’s test. All statistical analyses were performed in SPSS 15.0 (SPSS Inc., USA).

## Additional Information

**How to cite this article**: Chen, X. *et al*. The combined effects of sediment accretion (burial) and nutrient enrichment on the growth and propagation of *Phalaris arundinacea. Sci. Rep.* **7**, 39963; doi: 10.1038/srep39963 (2017).

**Publisher's note:** Springer Nature remains neutral with regard to jurisdictional claims in published maps and institutional affiliations.

## Figures and Tables

**Table 1:** Summary of the influence of each term (*F*-value) in a general linear model (GLM) testing the interacting influence of experimental sedimentation depth and nutrient enrichment on the growth of *Phalaris arundinacea*.

Effect	Biomass accumulation	Shoot mass ratio	Root mass ratio	Rhizome mass ratio	No. of ramets	No. of rhizomes	No. of buds	Ramet length	Rhizome length	Spacer length
Burial depth (B)	0.93^ns^	0.71^ns^	0.10^ns^	0.33^ns^	0.44^ns^	2.25^ns^	0.81 ^ns^	.82^ns^	1.05^ns^	0.38^ns^
Nutrient level (N)	51.36***	11.59**	45.51***	0.01^ns^	62.99***	33.71***	0.56 ^ns^	46.40***	6.45^*^	5.76*
B × N	0.11^ns^	0.54^ns^	3.83*	0.28^ns^	4.56*	1.02^ns^	0.83 ^ns^	2.94^ns^	0.18^ns^	1.69^ns^

***P < 0.001; **P < 0.01; *P < 0.05; ns *P* > 0.05.
